# Large spontaneous subcapsular hematoma of the liver: a rare case report

**DOI:** 10.11604/pamj.2019.32.16.17083

**Published:** 2019-01-10

**Authors:** Abdulrahman Al Tamimi, Awad Ali Alawad

**Affiliations:** 1 College of Medicine, King Saud Bin Abdulaziz University for Health Sciences, Riyadh, Saudi Arabia; 2 Department of Hepatobiliary Surgery and Organ Transplantation, King Abdulaziz Medical City, National Guard, Riyadh, Saudi Arabia

**Keywords:** Spontaneous, subcapsular hematoma, liver

## Abstract

Spontaneous subcapsular hematoma of the liver is an extremely rare but potentially life-threatening condition. We report a case of subcapsular hematoma of the liver without any apparent lesion and in the absence of coagulopathy or trauma. A CT scan of the abdomen demonstrated a huge subcapsular hematoma around the liver. The patient was treated conservatively and was discharged home after one week. Up to our knowledge, this is one of the very few reported cases of a spontaneous subcapsular hepatic hematoma.

## Introduction

The spontaneous subcapsular hematoma of the liver is very rare. There are only a few reported cases in the literature. Most reported cases of liver hematoma often occur during pregnancy as part of severe preeclampsia and HELLP syndrome [1]. The other causes may be due to rupture of hepatocellular carcinoma, adenoma, focal nodular hyperplasia or hemangioma [2-5]. Idiopathic spontaneous subscapular hematoma is a rare and often fatal condition. We are only aware of few spontaneous cases that have been reported previously.

## Patient and observation

**Figure 1:**
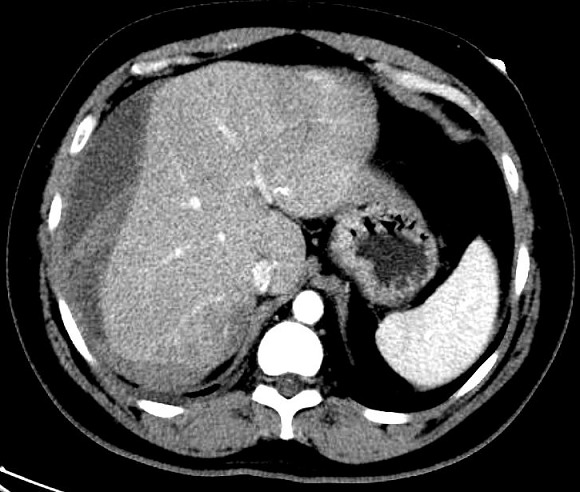
Abdominal computed tomography showing 16 x 10 x 3 cm large hepatic subcapsular hematoma and no focal lesion

**Figure 2:**
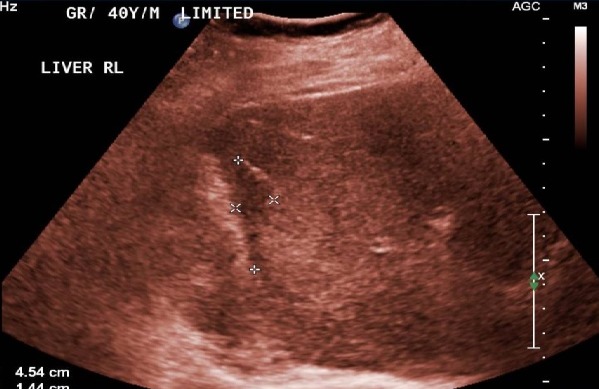
Sonography showing near complete resolution of the hematoma

A 40-year-old male, presented to the emergency department with sudden onset of right upper quadrant. The pain was severe, constant in nature, radiating to the right shoulder and aggravated by movement. Associated symptoms included nausea and one episode of vomiting. His medical history is unremarkable. The patient was an ex-smoker (stopped at the age of 30) and denied drinking alcohol. On physical examination, the patient looked ill and was not pale or jaundiced. He was hemodynamically stable with a heart rate of 104 and a blood pressure of 136/84 mm Hg. Oxygen saturations were 97% on room air and he was afebrile. There was no evidence of jaundice or lymphadenopathy. His abdomen was not distended, and no signs were elicited on palpation, suggestive of peritonitis. Bowel sounds were audible. Neurological, respiratory and cardiovascular examinations were normal. Laboratory results demonstrated hemoglobin 133 g/L, white cell count 8.5×109/L, platelets 188×109/L. The patient had a normal renal, liver and coagulation screen. An abdominal ultrasound revealed fluid collection around the right and left liver lobes suggestive of a hematoma. The CT scan of the abdomen showed 16 x 10 x 3 cm large hepatic subcapsular hematoma and no focal lesion (Figure 1). A diagnosis of hepatic subcapsular hematoma was made. He was managed conservatively, as he was stable, with complete bed rest, prophylactic antibiotics and analgesics. He didn't require any blood transfusion. The patient's symptoms resolved with conservative management and remained hemodynamically stable during admission. He was discharged home one week later, clinically back to his baseline and pain free. He is asymptomatic during follow-up visits till a period of 6 months, his serial sonographic scans show gradual decrease in the size of the hematoma. A follow up ultrasonography was done after 6 months and showed near complete resolution of the hematoma (Figure 2).

## Discussion

Spontaneous hepatic hematoma without underlying liver diseases is an uncommon entity. It represents an accumulation of blood between the capsule of Glisson and the liver parenchyma and frequently located around the right lobe (about 75 % of cases) [6]. The underlying cause of hepatic hematoma is varied. It has been frequently described in pregnancy as a complication of pre-eclampsia [7]. Other common causes include liver trauma and iatrogenic injuries following endoscopic retrograde cholangiopancreatography, biliary surgery and liver biopsy. Finally, it can also occur as a result of the underlying liver lesions such as liver neoplasms, coagulopathies and vasculitides. Our case is unusual as none of the aforementioned causes were present. The necessity of an early and accurate diagnosis in subcapsular hepatic hematomas derives from the modern trend of treating them conservatively, especially when the patient is hemodynamically stable. On the CT imaging, the hematoma typically looks like a lenticular, ellipsoid, perihepatic collection with a density that on the non-enhanced exam depends on the “age” of the hematoma. Acute hematomas are typically hyperdense (40-60 HU), due to the high protein content [8]. The density decreases in time due to the progressive lysis of hemoglobin becoming hypodense in the chronic phase. Although rare, they do have the potential to rupture; this can rapidly lead to haemodynamic instability and death. Therefore, a period of close monitoring is required in the acute setting. There should be a low threshold for radiological or surgical intervention, particularly if there is haemodynamic compromise or in the presence of a rapidly expanding hematoma associated with drop in hemoglobin. Firstly, an arterial embolization performed by interventional radiology should be attempted. Surgery should be considered where either embolization has failed or the haemodynamic instability is life-threatening. However, as in the case we have reported, the majority of non-ruptured spontaneous hepatic hematomas in haemodynamically stable patients can be managed non-operatively.

## Conclusion

In conclusion, spontaneous liver subcapsular hematoma can be observed in hemodynamically stable patients and it is a real challenge for surgeons.

## Competing interests

The authors declare no competing interests.
